# How meaningful are risk determinations in the absence of a complete dataset? Making the case for publishing standardized test guideline and ‘no effect’ studies for evaluating the safety of nanoparticulates versus spurious ‘high effect’ results from single investigative studies

**DOI:** 10.1088/1468-6996/16/3/034603

**Published:** 2015-05-05

**Authors:** David B Warheit, E Maria Donner

**Affiliations:** 1 Chemours Company, Wilmington, DE, USA; 2 DuPont Haskell Global Centers for Health & Environmental Sciences, Newark, DE, USA

**Keywords:** guideline studies, nanoparticles, no-effect studies, standardized studies, titanium dioxide, oral toxicity studies, biokinetic studies

## Abstract

A recent review article critically assessed the effectiveness of published research articles in nanotoxicology to meaningfully address health and safety issues for workers and consumers. The main conclusions were that, based on a number of flaws in study designs, the potential risk from exposures to nanomaterials is highly exaggerated, and that no ‘nano-specific’ adverse effects, different from exposures to bulk particles, have been convincingly demonstrated. In this brief editorial we focus on a related tangential issue which potentially compromises the integrity of basic risk science. We note that some single investigation studies report specious toxicity findings, which make the conclusions more alarming and attractive and publication worthy. In contrast, the standardized, carefully conducted, ‘guideline study results’ are often ignored because they can frequently report no adverse effects; and as a consequence are not considered as novel findings for publication purposes, and therefore they are never considered as newsworthy in the popular press. Yet it is the Organization for Economic Cooperation and Development (OECD) type test guideline studies that are the most reliable for conducting risk assessments. To contrast these styles and approaches, we present the results of a single study which reports high toxicological effects in rats following low-dose, short-term oral exposures to nanoscale titanium dioxide particles concomitant with selective investigative analyses. Alternatively, the findings of OECD test guideline 408, standardized guideline oral toxicity studies conducted for 90 days at much higher doses (1000 mg kg^−1^) in male and female rats demonstrated no adverse effects following a very thorough and complete clinical chemical, as well as histopathological evaluation of all of the relevant organs in the body. This discrepancy in study findings is not reconciled by the fact that several biokinetic studies in rats and humans demonstrate little or no uptake of nanoscale or pigment-grade TiO_2_ particles following oral exposures. We conclude that to develop a competent risk assessment profile, results derived from standardized, guideline-type studies, and even ‘no effect’ study findings provide critically useful input for assessing safe levels of exposure; and should, in principle, be readily acceptable for publication in peer-reviewed toxicology journals. This is a necessary prerequisite for developing a complete dataset for risk assessment determinations.

## Introduction

A recent review article by Krug [1] evaluated the efficacy and consistency of published nano-safety research papers for assessing health risks to humans, analyzing the strengths and weaknesses of over 10 000 publications. The Krug publication focused on aspects of human health effects or biological endpoints in animals, or cell cultures, as they pertained to the potential hazards of exposures to nanoparticles. This assessment was limited in scope but included studies on relevant *in vivo* uptake pathways related to the three most common routes of exposure—namely, via the respiratory tract (inhalation), gastrointestinal tract (GI tract, oral) or skin (dermal). The main conclusions drawn were that despite small degrees of biokinetic translocation following initial sites of airborne deposition (respiratory tract) or digestion (GI tract); adverse systemic effects due to secondary organ exposures are rare and much less of a concern than would be indicated by the results of high and often excessive dose exposures, common to many experimental studies. Moreover, as previously concluded by Donaldson and Poland [2], no ‘nano-specific’ effects are known to occur versus those reported with larger bulk particulate materials of identical composition. Perhaps more provocatively, Krug [1] concluded that many of the flawed designs employed in experimental toxicity studies inadvertently serve to generate questionable data which can give rise to erroneous conclusions regarding the toxicity of nanomaterials.

Krug also identified a number of significant limitations in scientific approaches for nanotoxicology studies pertaining to the topic of shortcomings of experimental or mechanistic-based studies for assessing human risks. It is apparent that one of the most obvious experimental design deficiencies results from a lack of robust physicochemical characterization of the nanomaterial test materials.

Our focus in this editorial relates, however, to other types of significant shortcomings and limitations in the experimental designs of nanotoxicology studies. These are evidenced by a number of fundamental issues including: high (nonrelevant) doses employed; a dearth of time course studies to gauge the sustainability of any measured responses; and a paucity of reference-type (i.e., control) samples to better interpret study findings. In addition, limited or questionable endpoints are often being measured, frequently selected based upon convenience or specific expertise in the laboratory, rather than because they are relevant safety assessment endpoints. Unfortunately, the absence of thoughtful planning of experimental designs may easily result in the promulgation of misleading conclusions about the effects of nano-particulates in biological systems.

Hypothesis-driven, experimental-type study results can provide significant value in postulating a mechanism of action, or in facilitating more insightful estimations of temporal events related to the development of pathological sequelae (e.g., adverse outcome pathways). Nonetheless, it is the standard practice for studies to follow the test guidelines (TGs) of the Organization for Economic Cooperation and Development (OECD), which provide the most compelling assessments of the hazards of a particular test material. These are the most widely used TGs specifically developed by OECD [3] for data generation and are commonly used for the objective of establishing safe exposure limits for a given form of a chemical substance, including particulates. It is not surprising that targeted experimental-type studies are more likely to produce new and/or (sensational) ‘unexpected’ findings. However, the pertinence of these results can rarely be reliably reproduced by independent investigators. Moreover, the novelty of the experimental findings are viewed as more newsworthy (often indicating more hazard than expected), and thus more attractive for publication, than to the more robust datasets generated from standardized guideline toxicity studies which report few if any novel effects and are often not considered publication-worthy. This creates a skewed bias favoring precautionary-type opinions based on singular studies which may exaggerate the hazards while excluding the substantial database demonstrating the safety of the materials. To illustrate this point, we briefly discuss some examples.

Tassinari *et al* [4] reported that oral exposures of rats to nanoscale titanium dioxide (TiO_2_) particles at 1 or 2 mg per kg body weight per day (mg kg^−1^ bw day^−1^) for 5 days produced a variety of adverse effects that are described in more detail below. In contrast to these reported findings, the results [5] of a subchronic 90-day oral toxicity study in rats conducted according to OECD TG 408 [6] with pigment-grade TiO_2_ test particles in rats demonstrated no adverse effects at doses up to 1000 mg kg^−1^ bw day^−1^—a differential dose of three orders of magnitude (compared to the Tassinari study)! Apart from these disparate oral toxicity results, numerous biokinetic study findings have reported negligible or no uptake of either nanoscale or pigment-grade TiO_2_ particles from the GI tract to the systemic circulation following acute or subchronic oral exposures in rats (Cho *et al* [7], Geraets *et al* [8], Himmelstein *et al* [9], MacNicoll *et al* [10]); as well as after acute oral dose exposures in human volunteers (Jones *et al* [11]).

How to reconcile this discrepancy? Many of the ‘no-effect’ OECD guideline studies are never published in the open literature, partly based on commercial confidentiality clauses, but in large part, due to the lack of novelty of findings. Therefore they unfortunately do not become part of the retrievable toxicology database. However, these hazard assessment studies (regardless of the outcome—positive or negative) represent the most valuable studies for evaluating human risks, because they are conducted according to Good Laboratory Practices (GLP) and fulfill a specific requirement according to mutually agreed upon standards.

Next, we describe the results from current biokinetic and toxicity studies for nanoscale and pigment-grade TiO_2_ following oral administration of fine and/or nanoscale test particles. The take-home points for this example are: (1) that the majority, if not all of the referenced biokinetic/pharmacokinetic studies of gastrointestinal uptake of either fine or nanoscale TiO_2_ particles in humans or rats following oral administration, have demonstrated negligible or no measurable uptake from the GI tract into the blood circulation; (2) a subchronic 90-day, high-dose oral toxicity study with TiO_2_ particles in rats demonstrated no adverse effects.

Below is a brief description of studies germane to the issue of uptake and toxicity of titanium dioxide particulates following oral exposures.

• A repeated 28-day oral gavage study in male rats with two different forms of pigment grade TiO_2_ particles. Male rats dosed with 24 000 mg kg^−1^ bw day^−1^ for 28 days (OECD TG 407) showed no adverse TiO_2_-related effects on clinical pathology parameters, organ weights, or gross/microscopic pathology endpoints. The NOAEL (no observable adverse effect level) was 24 000 mg kg^−1^ bw day^−1^ for male rats, the only dosages tested [12].
• A 90-day oral gavage study in rats with pigment grade TiO_2_ particles. Male and female rats were dosed with up to 1000 mg kg^−1^ bw day^−1^ (OECD TG 408). The results demonstrated that there were no adverse TiO_2_-related effects on clinical pathology parameters, organ weights or gross/microscopic pathology endpoints. The NOAEL for male and female rats was 1000 mg kg^−1^ bw day^−1^, the highest dosage tested [5].
• Human *in vivo* and *in vitro* studies on GI tract absorption of pigment grade and nanoscale TiO_2_ particles. Nine volunteers received a 5 mg kg^−1^ bw single oral dose of TiO_2_ dispersed in water. Three different studies were conducted with particle sizes of 15 nm (nanoscale), 100 nm (nano), and <5000 nm (pigment grade) TiO_2_. Urine samples were collected in timed collections over a 4-day period (over a time range of 24 h before dosing and 72 h post-dosing) and analyzed for titanium content after hydrolysis. Blood samples were collected before dosing and at 2, 4, 24 and 48 h post- dosing and analyzed for titanium content, full blood count and liver function tests. These human studies demonstrated that very little uptake occurred through the intestine for any of the TiO_2_ particle sizes tested. Moreover, the results of complementary *in vitro* studies in simulated gastric fluid indicated that all of the TiO_2_ formulations agglomerated and this action could be considered a ‘mechanistic contributor’ for the lack of uptake of particles from the GI tract to the systemic blood circulation [10].
• Similar methodologies and results were reported for *in vivo* and *in vitro* biokinetic studies investigating GI absorption of nanoscale and pigment grade TiO_2_ in rats following oral exposure to two forms of nanosized and one pigment-grade TiO_2_ test material. The findings of the *in vivo* component of the study demonstrated that oral administration of 5 mg kg^−1^ bw of TiO_2_ nanoscale or larger particles did not lead to any significant translocation of TiO_2_ (measured as titanium) from the gastrointestinal tract to either blood, urine or to various organs in rats at any of the time intervals studied over a 96 h post-administration period. Similar to the findings with humans, correlative *in vitro* study results demonstrated agglomeration of TiO_2_ particles in simulated gastric fluids. The authors concluded that the overall evidence from both *in vivo* and *in vitro* studies did not support that oral ingestion of nano- or larger-sized particles of TiO_2_ via food would result in any significant internal exposure of the consumer to the nanoparticles. The fate of dietary, ingested TiO_2_ nanoparticles results in the likely excretion in the faeces [9].
• In a recent pharmacokinetic screening study as per OECD TG 417, Himmelstein *et al* [8] assessed the uptake and subsequent systemic exposure following oral administration of either robustly characterized nanoscale or pigment-grade TiO_2_ particles in rats. The study included TiO_2_ analysis in blood, liver, urine, and faeces following single oral intubation of 500 mg kg^−1^ bw day^−1^ as either nano or pigment-grade TiO_2_ suspended in water. Collectively, both TiO_2_ test samples demonstrated very low potential for absorption and systemic exposure with nearly total quantitative recovery of the administered dose in the faeces. The results demonstrated the absence of oral uptake for nanosized or pigment grade TiO_2_ particles.
• Cho and coworkers [6] reported on the comparative absorption, distribution, excretion and toxicity of nano zinc oxide (ZnO) (doses up to 537 mg kg^−1^ bw day^−1^) or nanoscale TiO_2_ particles (doses up to 1042 mg kg^−1^ bw day^−1^) after repeated daily oral administration in rats (13 weeks). At the end of the study the concentrations of Zn in the blood of the ZnO-treatment groups demonstrated a clear dose-response relationship for blood concentrations. This pattern was not measured in TiO_2_ treated rats. The distribution patterns of Ti and Zn in the liver, spleen, kidney, and brain were measured using ICP-MS. Ti concentrations in the TiO_2_-treatment groups showed no significant increase in the sampled organs compared with the vehicle control group.
• However, in complete contrast to the results of the other presented studies, Tassinari *et al* [4] exposed rats to TiO_2_ nanoparticles (i.e., 1, and 2 mg kg^−1^ bw day^−1^ oral exposures) for 5 days. The investigators reported particle uptake in the spleen and ovaries but did not demonstrate translocation of particles from the GI tract into the blood circulation (as a prerequisite to their disposition into secondary organs). The adverse findings included histopathological effects in the thyroid, adrenals, ovaries, uterus, testes, and spleen.

These examples demonstrate how a robust data set generated by numerous pre-defined structured studies is discounted, based on lack of observed effects reported from studies with numerous undocumented variables and departure from conventional scientific principles only because of a predetermination that a test material must be hazardous. We need to remember that the OECD Principles of GLP and corresponding TG studies were developed to advance the quality and validity of test data utilized to establish the safety of the materials tested. The GLP concept represents a framework which encourages a set of deliberate procedures under which the conditions for planning and implementing laboratory studies are carefully documented and promulgated. The mutually agreed upon OECD TGs are required to be followed by test facilities conducting studies to be submitted to national authorities for evaluation of chemicals [3].

Accordingly, it seems reasonable to conclude that data from OECD guideline GLP studies should be viewed as reliable when no hazard is detected. In the event that the results of a particular study are not novel and show no effects (e.g., the findings of a 90-day oral toxicity study with TiO_2_ discussed above [5]), then the prospects of publishing the results have become somewhat problematic. On the other hand, a surprising finding, such as referred to in the Tassinari *et al* study [4]—with a dose range of three orders of magnitude less (e.g., 1–2 mg kg^−1^ bw day^−1^ versus 1000 mg kg^−1^ bw day^−1^), and a exposure duration of a 5-day versus a 90-day study—should require confirmation by independent investigators.

Indeed, one of these aforementioned studies was conducted according to strict OECD guidelines, while the other study was of an experimental nature. One of the aforementioned studies was implemented with the intention of submitting the results to national or international regulatory authorities; while the other study-type perhaps was developed to generate testable hypotheses. It seems clear that data which are developed for safety studies and utilized for risk assessment purposes should necessarily follow some form of a regulatory framework. Furthermore, because standardized guideline-driven studies which produce ‘no significant toxicological effects’ are often not viewed as novel or ‘newsworthy’—they have a reduced opportunity to be published in the peer review literature. Yet, ironically, these types of studies, regardless of the end results, may provide some of the most valuable datasets in assessing human health risks.

In this particular case, we have assessed the potential risks of oral exposures to nanoscale or pigment-grade TiO_2_ particles (particularly in food components). We have demonstrated that a careful review of the existing literature in exposed humans and rats clearly demonstrates a lack of significant biokinetic uptake from the GI tract to systemic blood circulation and corresponding secondary organs such as the liver and spleen. In addition, OECD guideline oral toxicity and pharmacokinetic studies demonstrate that oral doses in rats up to 1000 mg kg^−1^ bw day^−1^ do not produce adverse toxicological effects. In contrast, it has been reported in some experimental oral toxicity studies in rats at very low doses for 5 days, that exposures to nanoscale TiO_2_ particles have produced uptake (although not demonstrated in the blood circulation) and a variety of toxicity findings; thereby contravening the bulk of the reported biokinetic and toxicity studies conducted under guideline-type procedures. As a consequence, it is suggested that the results from the independent studies be confirmed by independent investigators and that guideline studies should be considered for publication in the peer-review literature, as these types of studies (because of the attention to quality procedures) should have a greater influence and enhanced credibility with respect to the development of safety assessments for humans.

In conclusion, there are three take-home messages presented herein: (1) standardized, guideline studies should be employed for conducting risk assessment determinations as well as for setting occupational exposure levels; (2) adverse-effects, experimental-type studies may be necessary and useful for investigating potential mechanisms of action or generating testable hypotheses, but have significant limitations for hazard or risk evaluations; (3) results derived from standardized, guideline-type studies—and even ‘no-effect’ study findings provide valuable input for determining safe levels, and should, in principle, be readily acceptable and are critical for submission to peer-reviewed, toxicology and health effects journals.

## Disclosure

The authors are employed by two companies (DuPont [EMD] and Chemours [DBW]) that make and commercialize pigment-grade TiO_2_ particles.
